# High Diversity of RNA Viruses in Rodents, Ethiopia

**DOI:** 10.3201/eid1812.120596

**Published:** 2012-12

**Authors:** Yonas Meheretu, Dagmar Čížková, Jana Těšíková, Kiros Welegerima, Zewdneh Tomas, Dawit Kidane, Kokob Girmay, Jonas Schmidt-Chanasit, Josef Bryja, Stephan Günther, Anna Bryjová, Herwig Leirs, Joëlle Goüy de Bellocq

**Affiliations:** Author affiliations: Academy of Sciences of the Czech Republic Institute of Vertebrate Biology, Brno, Czech Republic (Y. Meheretu, D. Čížková, J. Těšíková, J. Bryja, A. Bryjová, J. Goüy de Bellocq);; Mekelle University, Mekelle, Ethiopia (Y. Meheretu, K. Welegerima, Z. Tomas, D. Kidane, K. Girmay);; University of Antwerp, Antwerp, Belgium (Y. Meheretu, H. Leirs, J. Goüy de Bellocq);; Bernhard Nocht Institute for Tropical Medicine, Hamburg, Germany (J. Schmidt-Chanasit, S. Günther)

**Keywords:** RNA, viruses, rodents, Ethiopia, hantavirus, arenaviruses, Mobala virus, Arenaviridae, murine, Murinae, bunyaviruses, Bunyaviridae, RNA viruses

## Abstract

We investigated synanthropic small mammals in the Ethiopian Highlands as potential reservoirs for human pathogens and found that 2 rodent species, the Ethiopian white-footed mouse and Awash multimammate mouse, are carriers of novel Mobala virus strains. The white-footed mouse also carries a novel hantavirus, the second Murinae-associated hantavirus found in Africa.

Most emerging infectious diseases of humans or domestic animals are zoonoses, and among emerging pathogens, RNA viruses are highly represented (1). The synanthropic nature of some rodent species makes them important reservoirs of RNA viruses pathogenic to humans, such as hantaviruses (e.g., Seoul virus in black and Norway rats worldwide) and arenaviruses (e.g., Lassa virus in the multimammate mouse in western Africa or lymphocytic choriomeningitis virus in the house mouse worldwide). In Africa, members of the rodent genera *Mastomys* and *Arvicanthis* are linked to human activity; these rodents are widespread throughout sub-Saharan Africa and are crop pests and zoonotic reservoirs for human pathogens. Histories of synanthropy are likely longest for rodents in areas of early human sedentism, making RNA virus richness in early centers of domestication such as the Ethiopian Highlands of particular interest.

Hantaviruses (family *Bunyaviridae*) are RNA viruses primarily carried by rodents and soricomorphs (shrews and moles), although 2 new species have recently been described in bats (2,3). Arenaviruses (family *Arenaviridae*) are primarily rodent-borne RNA viruses. Members of both genera can cause life-threatening diseases in humans: arenaviruses cause hemorrhagic fevers in the Americas and Africa, and hantaviruses cause hemorrhagic fever with renal syndrome in Asia and Europe and hantavirus cardiopulmonary syndrome in the Americas. In Africa, only Lassa and Lujo arenaviruses are known to be highly pathogenic to humans. In contrast, hantaviruses have not yet been found to cause life-threatening human diseases in Africa, but hantavirus-specific antibodies have been found in human serum samples from several countries in Africa (4,5). To investigate the role of synanthropic small mammals as potential reservoirs of emerging pathogens in Ethiopia, we sampled rodent and shrew species in areas near human habitations and screened them for hantavirus and arenavirus RNA.

## The Study

Small mammals from domestic and peridomestic areas were trapped during August–December 2010 in 2 high-altitude localities, Golgolnaele (13°52′N, 39°43′E, elevation 2,700 m) and Mahbere Silassie (13°39′N, 39°08′E, elevation 2,600 m), and in 1 lower-altitude locality, Aroresha (12°25′N, 39°33′E, elevation 1,600 m), in the Tigray region of the Ethiopian Highlands. Kidney samples preserved in RNAlater reagent (QIAGEN, Hilden, Germany) and stored at –80°C were used for total RNA extraction by using the NucleoSpin RNA II Kit (Macherey-Nagel, Düren, Germany). Samples were pooled in pairs by locality and host species. RNA was reverse transcribed by using random hexamers as primers. Screening for arenaviruses was performed by using a pan–Old World arenavirus PCR targeting the large (L) gene (6). Screening for hantaviruses was performed by using a nested PCR assay targeting the hantavirus L gene (7).

A total of 201 small mammals from 6 species were screened for arenaviruses and hantaviruses (Table). Among them, 1 Ethiopian white-footed mouse (*Stenocephalemys albipes*) from Golgolnaele and 2 Awash multimammate mice (*Mastomys awashensis*) from Aroresha were positive for arenavirus RNA; 10 white-footed mice from the 2 highland localities (6 from Golgolnaele, 4 from Mahbere Silassie) were positive for hantavirus RNA. Amplicons were purified and sequenced, and nucleotide sequences were aligned on the basis of the amino acid alignment. Phylogenetic analyses were performed on the nucleotide sequences by using a maximum-likelihood (ML) approach (8).

**Table:** Small mammal species screened for arenaviruses and hantaviruses, Ethiopian Highlands, August–December 2010*

Species	No. animals by locality and elevation			Total no. animals
	Aroresha, 1,600 m	Golgolnaele, 2,700 m	Mahbere Silassie, 2,600 m	
Ethiopian white-footed mouse (*Stenocephalemys albipes*)	0	33	23	56
Awash multimammate mouse (*Mastomys awashensis*)	16	1	1	18
*Mus* (*Nannomys*) sp. mice	11	20	2	33
Black rat (*Rattus rattus*)	37	2	5	44
African giant shrew (*Crocidura olivieri*)	6	2	17	25
Dembea grass rat (*Arvicanthis dembeensis*)	8	13	4	25
Total	78	71	52	201

*Species identification was confirmed by sequencing the partial mitochondrial cytochrome b gene (11). Representative sequences are available in GenBank (accession nos. JQ956464-JQ956479). Voucher specimens from representative rodents have been deposited at the Evolutionary Ecology group, University of Antwerp, and are available from the authors on request.

**Figure 1:**
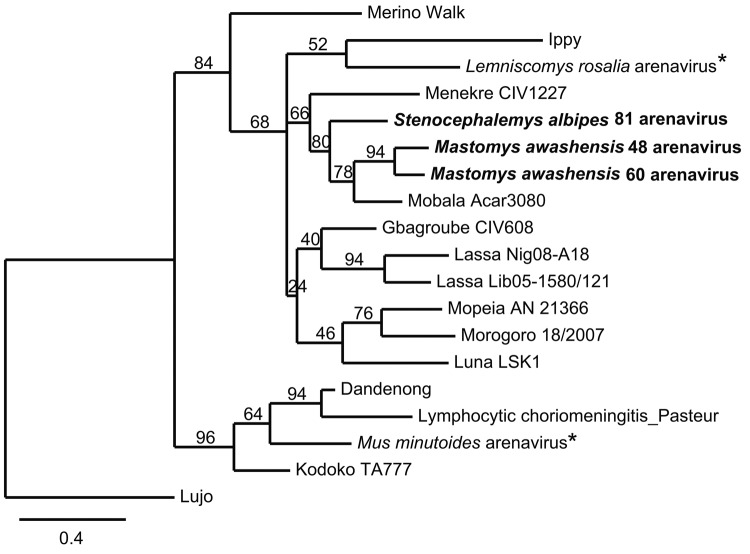
Maximum-likelihood tree of Old World arenaviruses showing the position of 3 arenaviruses (**boldface**; GenBank accession nos. JQ956481–JQ956483) found in kidney samples of Awash multimammate mice (*Mastomys awashensis*) and Ethiopian white-footed mice (*Stenocephalemys albipes*). The tree was constructed on the basis of analysis of partial sequences of the RNA polymerase gene; phylogeny was estimated by using the maximum-likelihood method with the GTR + I + Γ (4 rate categories) substitution model to account for rate heterogeneity across sites as implemented in the PhyML program (8). Lujo arenavirus was used as an outgroup. Numbers represent percentage bootstrap support (1,000 replicates). *Arenaviruses from Tanzania that have not yet been named (9). Scale bar indicates nucleotide substitutions per site. GenBank accession numbers of the virus strains: EU136039, GU830849, AY363902, EF179864, GU979511, GU481071, DQ868486, GU182412, FJ952385, AB586645, GU830863, GU078661, DQ328876, AY363904, EU914110, GU182413.

After sequencing of the 3 arenavirus-positive samples, 3 distinct arenavirus sequences were obtained, and an ML tree was constructed for these 3 arenavirus sequences and the partial L gene (340 bp) of representatives of Old World arenaviruses (Figure 1). The 3 sequences cluster with Mobala virus (80% bootstrap support), an arenavirus discovered in *Praomys* sp. in the Central African Republic in 1983 (10). However, the 3 sequences from Ethiopia are not monophyletic; the 2 sequences from multimammate mice cluster together (94% bootstrap support), but the sequence from the white-footed mouse from Golgolnaele is basal to the clade (Mobala + *M. awashensis* virus sequences), with the Menekre virus, found in *Hylomyscus* sp. in Guinea (11), as outgroup. The sequences from multimammate mice on average differ from those of Mobala virus and the sequence from the white-footed mouse by the same order of magnitude in terms of amino acids: 5.0 ± 2.1% and 5.9 ± 2.2%, respectively. The average amino acid difference between the sequence from the white-footed mouse and that from Mobala virus was 8.1 ± 2.6%. Therefore, these arenaviruses seem to be 2 strains of Mobala virus carried by 2 rodent species and found in 2 localities ≈250 km apart from each other and with an altitude difference of 1,100 m.

**Figure 2:**
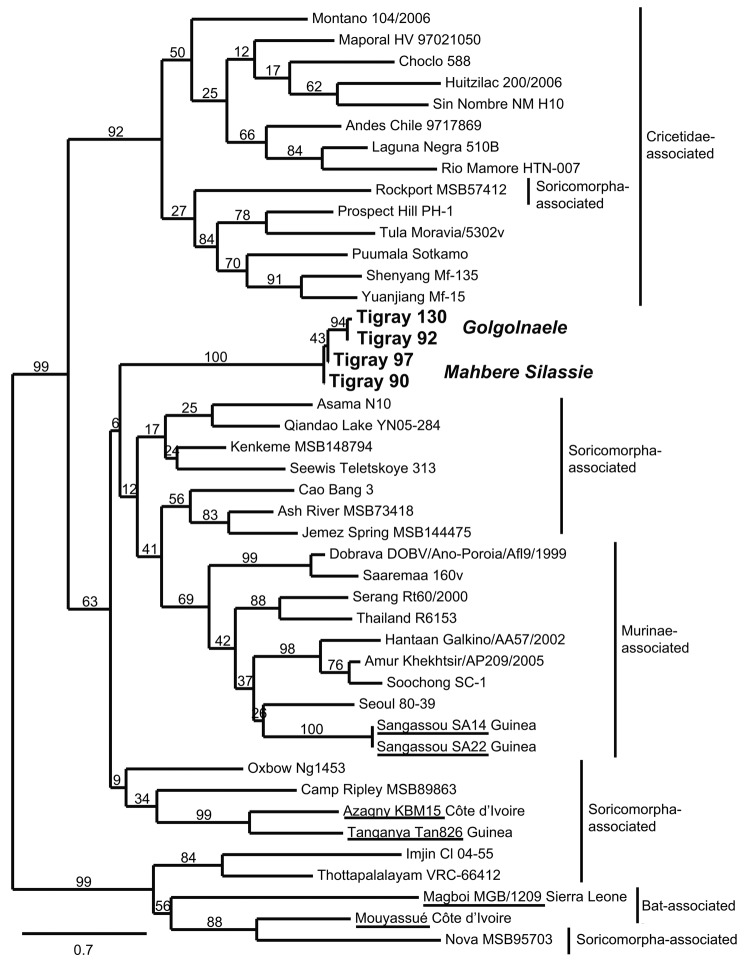
Maximum-likelihood tree of hantaviruses showing the position of the 4 sequences of Tigray hantavirus (**boldface**; GenBank accession nos. JQ956484–JQ956487) found in kidney samples of Ethiopian white-footed mice (*Stenocephalemys albipes*). The tree was constructed on the basis of analysis of partial sequences of the RNA polymerase gene; phylogeny was estimated by using the maximum-likelihood method with the general time reversible + I + Γ (4 rate categories) substitution model to account for rate heterogeneity across sites as implemented in the PhyML program (8). Numbers represent percentage bootstrap support (1,000 replicates). Underlining indicates hantaviruses found in Africa. Scale bar indicates nucleotide substitutions per site. GenBank accession numbers of the virus strains: AB620030, NC_003468, EU929078, EF619961, JF276228, EF540771, EF543525, EF397003, NC_005235, AB620033, JN037851, FJ170809, FJ170812, AB620108, EF641807, FJ593501, GQ306150, AF005729, EU788002, AB620102, FJ593498, FJ593497, EF646763, NC_005225, GU566021, FJ809772, HM015221, AJ410618, DQ268652, JQ082305, EU424336, NC_005238, AM998806, NC_005217, DQ056292, EF050454, JN116261, EU001330, AJ005637, JQ287716.

After sequencing of the 10 hantavirus-positive samples, 4 distinct hantavirus sequences were obtained, 2 from Golgonaele and 2 from Mahbere Silassie. Figure 2 shows the ML tree for these 4 sequences and the partial L gene (347 bp) of representatives of hantaviruses. The tree is not well resolved, and shrew- and mole-associated hantaviruses do not cluster. Two rodent-associated clades are supported: the previously known Murinae-associated hantaviruses (69% bootstrap support) and the Cricetidae-associated hantaviruses (92% bootstrap support, with 1 exception, Rockport, Soricomorpha-associated hantavirus). Although all 4 sequences were found in *S. albipes* mice, a Murinae species endemic to Ethiopia, they do not group with the Murinae-associated hantaviruses or with hantaviruses found in other African small mammals, such as bats (2,3) or shrews (12,13). The 4 sequences form a unique, divergent clade with the 2 sequences from Mahbere Silassie basal to the sequences from Golgonaele, which cluster together. The average amino acid difference between the sequences from Ethiopia and those from Murinae-associated hantaviruses was 27.0 ± 4.0%. Because the new amino acid sequences are at least 21.0 ± 4.0% divergent from those of other hantaviruses, we conclude that *S. albipes* mice are carrying a novel hantavirus. We propose the name Tigray virus for this virus because it was found in the Tigray region of Ethiopia. Additional genetic characterization, in particular of the small and medium segments, will be conducted to further clarify the evolutionary relationship of this virus within the hantavirus genus.

## Conclusions

Two rodent species living in close proximity to human settlements in Ethiopia are carriers of arenaviruses and hantaviruses. Recently, several new arenaviruses and hantaviruses have been described in small mammals in Africa, but no clear association with human diseases has been found (2,3,9,11–13). However, arenavirus and hantavirus infections are likely severely underreported because symptoms may resemble those of many other febrile infections (2). Investigating the presence of antibodies for Mobala virus and the proposed Tigray virus in humans in the Ethiopian Highlands is the next step in evaluating their pathogenicity. A recent study in Guinea showed that 2/68 patients with fever of unknown origin had antibodies for Sangassou hantavirus (5); a case of putative hantavirus disease (hemorrhagic fever with renal syndrome) was also reported in the Central African Republic (14). Hantavirus infections may thus be an unrecognized medical problem in Africa and deserve more attention.

In conclusion, our screening of 201 small mammals led to the identification of 2 novel strains of Mobala arenavirus and a novel hantavirus in 2 rodent species found in Ethiopia, *M. awashensis* and *S. albipes*. These rodents belong to the exclusively African Praomyini tribe (15), which hosts 5/11 arenaviruses (Lassa, Mopeia, Luna, Mobala, and Menekre viruses) and the only Murinae-associated hantavirus (Sangassou virus) described in Africa. Our results support a major role for Praomyini as hosts in the evolutionary history of arenaviruses and hantaviruses in Africa.
